# Targeting delayed healing tissues: immune reprogramming of adaptive immune cells by biomaterials

**DOI:** 10.1093/rb/rbag033

**Published:** 2026-03-05

**Authors:** Zhiyun Wu, Fengzhen Meng, Yuming Xiang, Xiaohua Pan, William W Lu, Shaoquan Bian, Fan Pan, Liping Ouyang, Xiaoli Zhao

**Affiliations:** Research Center for Human Tissues and Organs Degeneration, Shenzhen Key Laboratory of Marine Biomedical Materials, Institute of Biomedicine and Biotechnology, Shenzhen Institutes of Advanced Technology, Chinese Academy of Sciences, Shenzhen 518055, China; University of Chinese Academy of Sciences, Beijing 100049, China; Research Center for Human Tissues and Organs Degeneration, Shenzhen Key Laboratory of Marine Biomedical Materials, Institute of Biomedicine and Biotechnology, Shenzhen Institutes of Advanced Technology, Chinese Academy of Sciences, Shenzhen 518055, China; Research Center for Human Tissues and Organs Degeneration, Shenzhen Key Laboratory of Marine Biomedical Materials, Institute of Biomedicine and Biotechnology, Shenzhen Institutes of Advanced Technology, Chinese Academy of Sciences, Shenzhen 518055, China; College of Engineering, Southern University of Science and Technology, Shenzhen, Guangdong 518055, China; Institute of Clinical Translation and Regenerative Medicine, People’s Hospital of Baoan District, The Second Affiliated Hospital of Shenzhen University, 518101, Shenzhen, China; Research Center for Human Tissues and Organs Degeneration, Shenzhen Key Laboratory of Marine Biomedical Materials, Institute of Biomedicine and Biotechnology, Shenzhen Institutes of Advanced Technology, Chinese Academy of Sciences, Shenzhen 518055, China; Faculty of Pharmaceutical Sciences, Shenzhen University of Advanced Technology, Shenzhen 518107, China; Research Center for Human Tissues and Organs Degeneration, Shenzhen Key Laboratory of Marine Biomedical Materials, Institute of Biomedicine and Biotechnology, Shenzhen Institutes of Advanced Technology, Chinese Academy of Sciences, Shenzhen 518055, China; University of Chinese Academy of Sciences, Beijing 100049, China; Faculty of Pharmaceutical Sciences, Shenzhen University of Advanced Technology, Shenzhen 518107, China; Shanghai Key Laboratory of Flexible Medical Robotics, Tongren Hospital, Institute of Medical Robotics, Shanghai Jiao Tong University, Shanghai 200336, China; Research Center for Human Tissues and Organs Degeneration, Shenzhen Key Laboratory of Marine Biomedical Materials, Institute of Biomedicine and Biotechnology, Shenzhen Institutes of Advanced Technology, Chinese Academy of Sciences, Shenzhen 518055, China; University of Chinese Academy of Sciences, Beijing 100049, China; Faculty of Pharmaceutical Sciences, Shenzhen University of Advanced Technology, Shenzhen 518107, China

**Keywords:** tissue regeneration, adaptive immune system, biomaterials, immune reprogramming, physicochemical

## Abstract

The adaptive immune system plays a pivotal role in coordinating tissue regeneration. Effective tissue repair and functional recovery depend on precisely regulating adaptive immune cells, especially T and B lymphocytes, and their dynamic crosstalk with innate immune cells. The immunomodulatory effects of biomaterials on adaptive cells could be realized through their influence on innate immune cells. This presents a promising therapeutic strategy, in which biomaterials can be designed to modulate immune activity toward pro-regenerative pathways and enhance healing. This review summarizes current knowledge on the molecular mechanisms that distinguish efficient acute healing from pathological chronic or delayed repair processes. Special attention is given to how key physicochemical properties of biomaterials influence the behavior of immune cells, with a specific emphasis on modulating adaptive immune responses. Understanding these biomaterial-immune cell interactions would advance the fundamental understanding of immunomodulatory biomaterials and provide a rational framework for designing next-generation biomaterials with optimized properties. This approach would help harness the body’s intrinsic immune mechanisms to improve the outcomes of tissue regeneration. Future studies will refine these design principles to accelerate clinical translation.

## Graphical Abstract

**Graphical Abstract:**
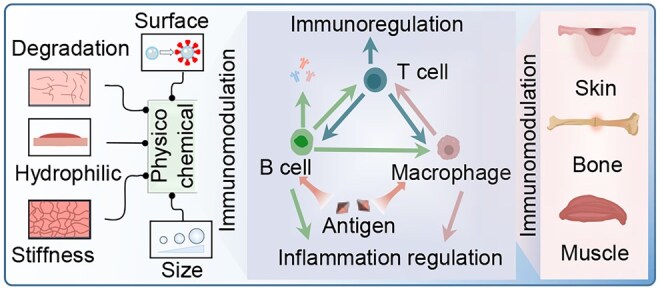

## Introduction

Tissue injury resulting from trauma, infections, or diseases presents a major global healthcare burden. A critical challenge is delayed healing often caused by persistent inflammation and dysregulation of the immune microenvironment, which can lead to non-healing or recurrent wounds [1, 2]. Affecting an estimated 1–2% of the global population, chronic wounds severely impair patients’ quality of life [3]. Defense and repair involve a coordinated response from both the innate immune system, which provides rapid, broad defenses against pathogens, and the adaptive immune system, which offers antigen-specific recognition and long-term protection [4, 5]. This interplay is essential for maintaining the body’s homeostasis. Therefore, the immunomodulatory microenvironment is increasingly recognized as crucial in regulating inflammation and tissue repair, which has attracted extensive research attention.

In recent years, immunomodulatory biomaterials have been demonstrated significant potential to accelerate healing and restore tissue function in tissue repair [6–8]. By elucidating the mechanisms of how biomaterial properties regulate the immune microenvironment, scientists can design materials with specific compositions and structures to target immunomodulation for therapeutic benefit [9, 11]. While past studies have largely explored biomaterial interactions with innate immunity [12–14], emerging evidence highlights the pivotal role of adaptive immune cells, especially T cells, in delayed tissue repair scenarios. Dysregulated T cell responses, such as excessive pro-inflammatory T helper cells activation or impaired regulatory T cell (Treg) function, have been linked to pathological outcomes [15, 16]. Therefore, this review summarizes the factors underlying delayed tissue healing and discusses how biomaterial physicochemical properties can be strategically engineered to modulate immune cells, with a dedicated focus on adaptive immunity. By emphasizing the modulation of T cell and B cell phenotypes and functions, this review aims to offer novel perspectives for developing immunologically guided biomaterials for hard-to-heal tissues.

## Overview of the immune system

Tissue repair course following injury is generally divided into four successive and overlapping phases: hemostasis, inflammation, proliferation, and remodeling [17, 18]. The initial hemostatic phase involves vessel constriction and platelet activation at the injury site. Undergoing aggregation and release reactions, platelets form a hemostatic platelet thrombus. This is followed by blood coagulation, where fibrinogen transforms into fibrin to form an intertwined network that further stops bleeding.

The inflammatory phase, peaking within 48–72 h after injury, is characterized by the infiltration of innate immune cells. Monocytes migrate toward the damaged area and phagocytose bacteria, necrotic tissues, cellular debris, etc. Beyond clearance, macrophages secrete a variety of inflammatory factors, orchestrating the early immune response and preparing for subsequent repair process [19]. While innate immunity dominates the early stage, adaptive immune cells become crucial in the later stages of wound healing. While elevated levels of CD8^+^ T cells can hinder tissue regeneration, CD4^+^ helper T cells facilitate the process, and Tregs help modulate inflammation by downregulating immune response [20]. Additionally, Th2 cells coordinate with Th17 cells contributing to angiogenesis and the transition of tissue repair into the proliferative phase, either directly or indirectly [21].

During the proliferative phase, new granulation tissue is generated, supported by robust angiogenesis that restores nutrient and oxygen supply [22]. In the final remodeling phase, collagen is continuously decomposed and resynthesized. Type III collagen is gradually replaced by type I collagen, cross-connecting type I collagen continues to increase, and tissue elasticity gradually increases [23]. Concurrently, vascular permeability returns to normal, and tissue properties gradually restore to pre-injury state.

**Figure 1:**
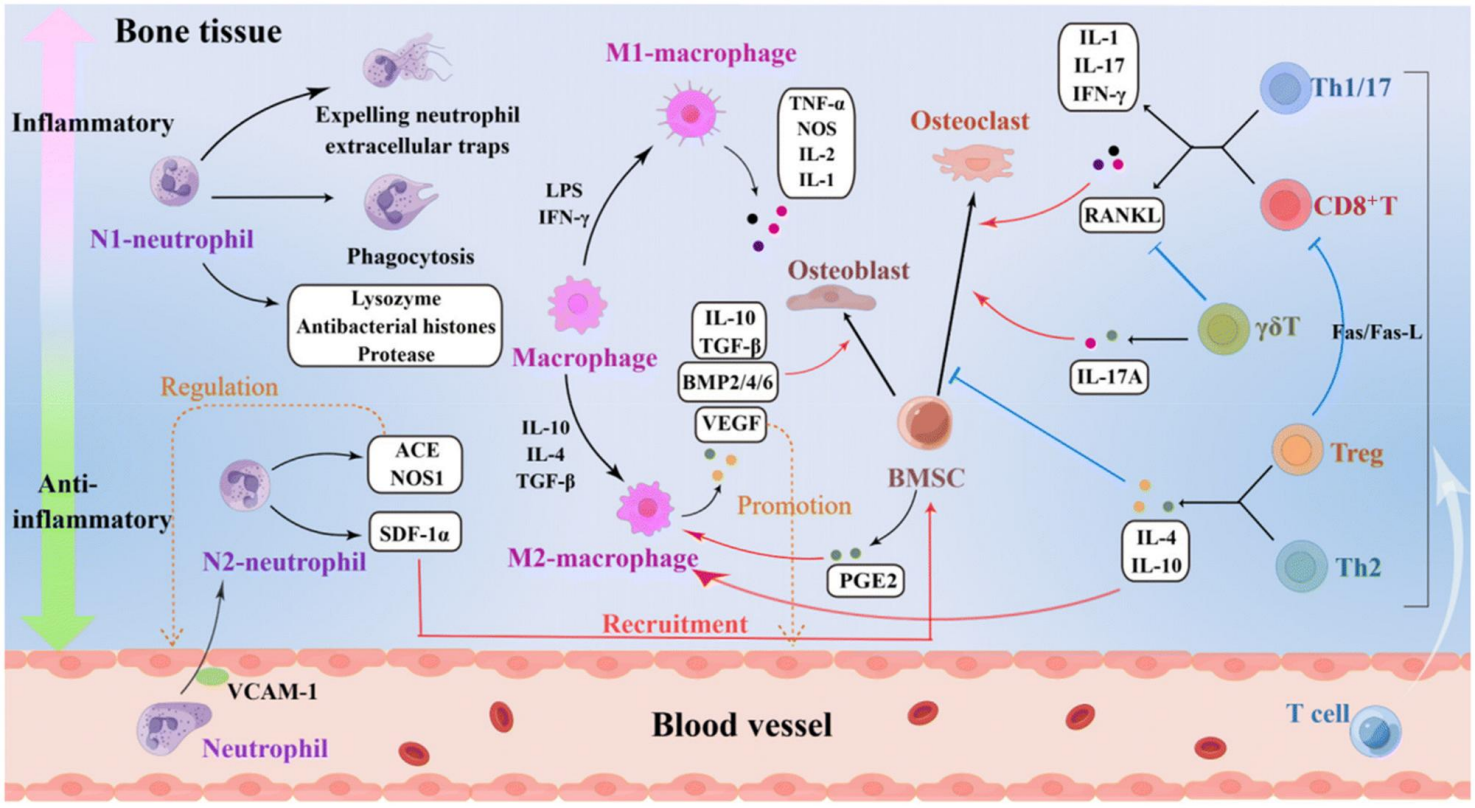
The dynamic interaction between immune cells and functional tissue cells during fracture repair. This schematic illustrates the coordinated cellular events that bridge the initial inflammatory response to subsequent bone regeneration. Following a fracture, various immune cell types are recruited to the injury site and undergo synchronized activation. This process aims to establish a pro-repair immune microenvironment that supports osteoblast activity and enhances the differentiation, thereby facilitating regeneration. Symbol codes are defined as follows: arrows indicate a promoting or enhancing effect. Blunt-ended horizontal lines indicate an inhibitory effect. Reproduced with permission from Ref. [24].

In summary, the immune system plays an important role in coordinating tissue healing, although the specific stages and the processes involved may vary in different tissues (Figure 1) [24].

### Underlying immune mechanisms of delayed tissue formation

**Figure 2:**
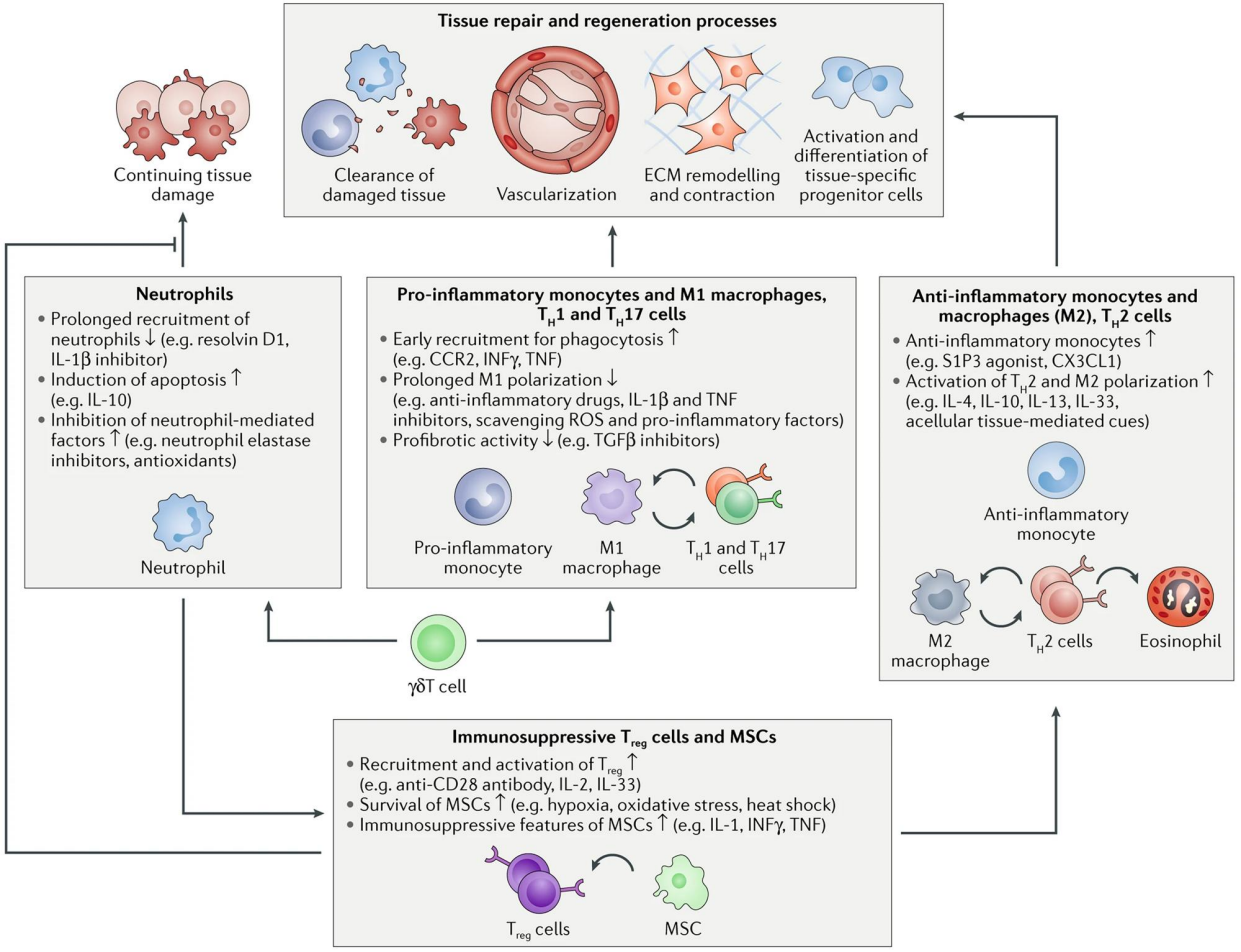
Factors contributing to delayed tissue healing and immunotherapeutic strategies for intervention. Tissue repair can be impeded by multiple factors, including hyper-inflammation, bacterial infections, and cellular senescence. The repair and regeneration process involves clearance of damaged tissue, vascularization, remodeling of extracellular matrix (ECM), as well as the proliferation and differentiation of stem and progenitor cells. These processes are orchestrated by various immune cells that play distinct roles at different stages of tissue repair following injury. The diagram depicts the involvement of key immune cell types in the tissue repair process and their functional effects. The immunomodulatory strategies to promote tissue repair include promoting the resolution of neutrophils, shifting macrophage polarization from pro-inflammatory M1 to pro-repair M2 phenotypes, modulating T helper cell responses, enhancing Treg recruitment and function, and utilizing immunosuppressive features of mesenchymal stromal cell (MSC). Reproduced with permission from Ref. [27].

The pathological mechanism by which acute wounds become non-healing remains unclear. Multifactorial stimuli lead to delayed tissue healing, including but not limited to, functionally impaired immune cells, a hostile microenvironment, cellular aging, microbial infection, and biofilm formation [25]. These factors lead to persistent inflammation [26], which will be discussed in this section [27] (Figure 2). The fundamental cause lies in the prolonged disruption of the immune environment, leading to abnormalities in both the number and function of immune cells. Notably, T cell differentiation and function are highly susceptible to this dysregulated microenvironment. For instance, studies have shown reduced effector T cell accumulation and T cell receptor (TCR) library diversity in diabetic patients [28, 29]. We have found that metabolic regulation by hypoxia-inducible factor 1-alpha (HIF-1α) could regulate Treg function and Th17/Treg ratio to influence inflammation environment [30]. Specifically, under hyperglycemic conditions, immune cells may become functionally impairment, compromising normal physiological functions and consequently delaying tissue healing. Metabolic disorders in cellular immunity thus represents another critical factor that cannot be overlooked [31]. Given the numerous and complexly interconnected factors influencing delayed tissue healing, and the fact that its underlying mechanisms remain incompletely understood, there is an urgent need for more in-depth and systematic research to uncover key pathways and regulatory networks.

### Contribution of immune cells to tissue healing

**Figure 3:**
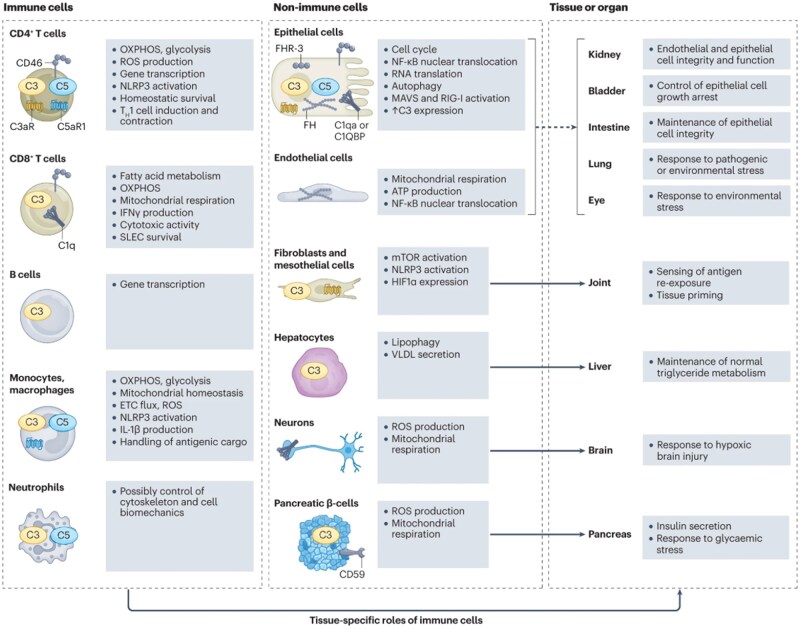
The functions and regulatory mechanisms of various cell types in different tissues and organs highlight the crucial role of immune cells in tissue repair. Immune cells (including CD4^+^ T cells, CD8^+^ T cells, B cells, monocytes, macrophages, neutrophils) coordinate with resident tissue-specific non-immune cells (such as epithelial, endothelial, fibroblast, hepatocyte, and neuron cells) across different tissues. Immune-metabolic conversion synchronizes the restart of parenchymal cell cycles, barrier epithelial repair, and organ-specific lipid redistribution, while preventing excessive fibrosis progression. Reproduced with permission from Ref. [40].

Tissue repair is a dynamic cascade of biological events, including the recruitment of immune cells, clearance of necrotic debris, cellular proliferation and differentiation, angiogenesis, and extracellular matrix (ECM) deposition and remodeling [32]. Immune cells are indispensable to this process, with their specific involvement shaped by the type of injury and the regenerative capacity of the tissue. In the early stages of injury, innate immune cells, particularly neutrophils and macrophages, are pivotal in recruiting additional immune cells and clearing cellular debris [23–37]. An appropriately regulated inflammatory response is beneficial for eliminating pathogens and cellular debris. As innate immunity plateaus, adaptive immune cells assume critical roles in regulating their activity and preserving tissue homeostasis, often through direct crosstalk with innate immune cells [38–40] (Figure 3).

Upon tissue injury, a spectrum of innate immune cells (e.g. macrophages, mast cells, neutrophils, langerhans cells, and natural killer (NK) cells), along with adaptive immune cells (T cells and B cells), are sequentially recruited to facilitate healing. Tissue-specific contexts further define these interactions. In skin tissue, keratinocytes, fibroblasts, and endothelial cells cooperate with various immune cells to promote skin repair [41, 42]. The healing of bone tissue involves chondrocytes, osteoblasts, osteoclasts, and mesenchymal stem cells (MSCs) to participate, while muscle repair involves non-immune cells, such as satellite cells and myofibroblasts [43, 44]. This diversity indicates the heterogeneity of cellular components involved in tissue repair. A detailed discussion of the immune cells that contribute to the healing process is presented below.

#### Innate immune cells

The innate immune system is a key driver of tissue repair. It detects damage-associated molecular patterns, triggering inflammatory responses that recruit neutrophils and macrophages. These cells subsequently release factors such as vascular endothelial growth factor (VEGF), transforming growth factor-β (TGF-β), and matrix metalloproteinases to coordinate angiogenesis, ECM remodeling, and stem cell activation to ultimately restore tissue structure and function.

##### Neutrophils

Neutrophils respond rapidly to pathogens by releasing reactive oxygen species and granules, forming neutrophil extracellular traps (NETs), particularly in response to larger pathogens [45, 46]. Neutrophils also secrete immunoregulatory cytokines and chemokines, such as interleukin-1 beta (IL-1β), IL-6, and CXCL8, which recruit additional neutrophils, macrophages, and T cells to the injury site. Furthermore, neutrophils have been shown to influence the behaviors of other immune cells, potentially promoting the polarization of macrophages toward an anti-inflammatory phenotype [19]. Notably, some studies have indicated that neutrophils can directly present antigens to T cells, thereby initiating adaptive immune responses [47]. Functionally distinct subsets of neutrophils are likely to drive specific responses during tissue regeneration [48]. Collectively, these findings underscore the essential role of neutrophils in skin injury responses, facilitating pathogen clearance and the release of anti-inflammatory cytokines, ultimately contributing to effective wound healing.

##### Macrophages

Macrophages are essential in skin regeneration, bone healing [49], and neovascularization [50]. Often classified into pro-inflammatory M1 and pro-repair M2 phenotypes based on *in vitro* characterization and functionality, they exhibit a remarkable degree of plasticity [51–53]. Notably, the sequential activation of these phenotypes can influence inflammation levels and tissue regenerative outcomes, making equilibrium between M1 and M2 macrophages essential [54]. In summary, macrophages facilitate tissue repair and revascularization by clearing pathogens and cellular debris, remodeling the ECM, and promoting angiogenesis [55, 56].

##### Other cells

Langerhans cells sense injury, migrate to lymph nodes to activate T cells, and secrete IL-6 and TGF-β to modulate inflammation and fibroblast activity. Mast cells exhibit a biphasic role, initial degranulation to initiate the inflammatory response, followed by factor secretion to finely tune vascular and collagen remodeling. Timely activation promotes healing, whereas sustained activation leads to chronic inflammation and fibrosis [57]. NK cells release pro-inflammatory cytokines like tumor necrosis factor-alpha (TNF-α) and interferon-gamma (IFN-γ), and can interact with other immune cells such as neutrophils and macrophages [58].

#### Adaptive immune cells

Recent advances in research have increasingly highlighted the significance of adaptive immune cells (T and B cells) in tissue repair [59]. T cells respond to various signals from innate immune cells by producing cytokines, thereby coordinating tissue repair processes. Most αβ T lymphocytes are resident T cells that maintain homeostasis in the skin tissue environment [60]. The γδ T cells play an important role in resisting infection. B cells not only produce antibodies in response to antigens but also secrete cytokines that modulate the activities of other immune cells [20]. However, the role of the adaptive immune system in tissue repair requires further exploration.

##### T cells

T cells can be broadly categorized into CD4^+^ helper cells and CD8^+^ cytotoxic cells, each fulfilling distinct physiological roles [20]. Although CD8^+^ T cells help clear necrotic tissue and activate macrophages, their overall influence has been observed to hinder tissue regeneration [61, 62]. CD4^+^ T subsets play divergent roles. Th2 cells cooperate with Th17 cells facilitating tissue regeneration and angiogenesis through secreting various regenerative factors (e.g. IL-4, IL-5, IL-17, and IL-21). Conversely, Th1 cells induce inflammation to eliminate intracellular pathogens. Tregs play a crucial role in moderating excessive immune responses and supporting tissue regeneration through the release of immunomodulatory molecules [63–68].

##### B cells

B cells function as antigen-presenting cells (APCs) [69]. Beyond their capability to produce antibodies, B lymphocytes significantly influence skin inflammation through cytokine secretion. After injury, B cell-derived IgG can enhance macrophage phagocytosis and pro-angiogenic functions [70]. Furthermore, under certain conditions, B cells can also adopt a regulatory phenotype that suppresses immune responses [71, 72]. Interestingly, both the presence and absence of B cells have been reported to positively affect wound healing [73, 74].

Thus, modulating adaptive immune cells has emerged as a crucial factor in determining outcomes related to tissue regeneration.

## Effects of the physicochemical properties of biomaterials on innate and adaptive immune cells

While acute wound management is typically straightforward and requires minimal intervention, chronic wounds present a major clinical challenge. Clinical approaches to chronic wounds encompass strategies such as debridement (including mechanical and autolytic methods), management of underlying pathologies, and moisture control [1]. Nevertheless, conventional treatments often fail to address the recurrent nature. Researchers aim to address this fundamental issue by modulating the wound microenvironment, which has prompted extensive investigations into potential biomaterial-based solutions. Therefore, there is a pressing need for effective therapies that enhance tissue repair and regeneration either by using biomaterials to promote endogenous repair mechanisms [46].

**Figure 4:**
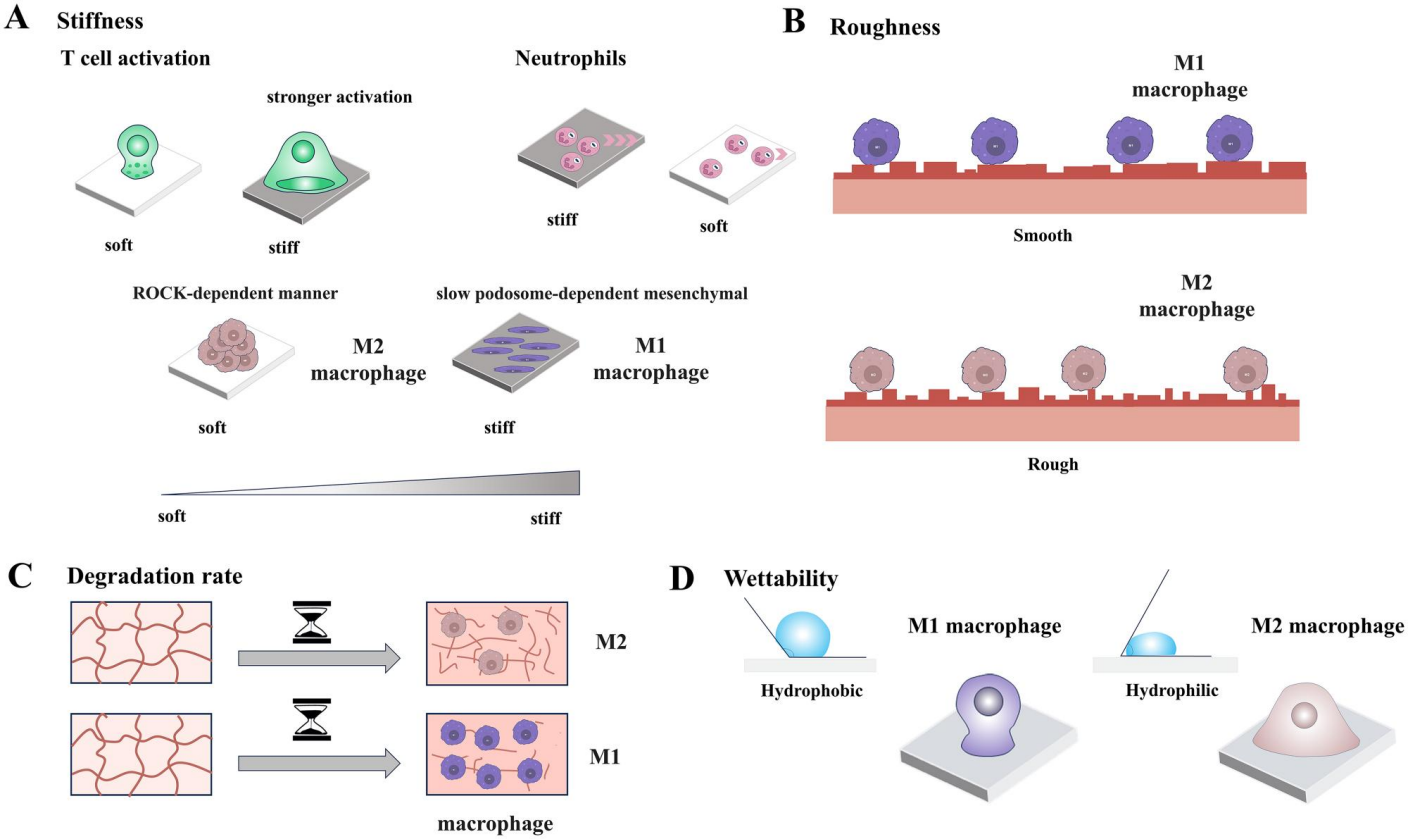
Modulation of immune cell function by altering biomaterial properties. This schematic summarizes how the material properties such as stiffness, roughness, degradation rate, and wettability, can be engineered to regulate immune cell function. (**A**) Stiffness: Harder materials generally enhance T cell activation. Neutrophils migrate faster on stiff matrices but spread more on soft ones. Stiffness also promotes M1-like macrophage polarization and aggregation, while soft biomaterials favor an anti-inflammatory state. (**B**) Roughness: Rougher surfaces of biomaterials tend to polarize macrophages toward an M2-like phenotype, while smoother surfaces promote a pro-inflammatory state. (**C**) Degradation rate: Rapidly degrading biomaterials often trigger strongest macrophage activation and a more severe inflammatory response due to the body’s inadequate ability of clearance. (**D**) Hydrophobicity: Hydrophobic surfaces tend to induce pro-inflammatory M1-like macrophage activation, whereas hydrophilic or neutral surfaces support an anti-inflammatory microenvironment.

Endogenous repair relies on mobilizing host immune cells to restore tissue homeostasis [75]. Notably, biomaterials hold significant promise for reconstructing the injury microenvironment with broad applications in tissue engineering [76]. Their efficacy depends on physicochemical and degradation properties [77], which dynamically interact with the host to shape immune response (Figure 4). Numerous studies have elucidated the biological processes associated with foreign-body response [78–82]. In recent years, various biosensors for detecting and analyzing immune cell dynamics have also been continuously refined, gradually becoming indispensable tools [83]. Additional studies indicate that optogenetic tools hold the potential for precisely regulating TCR activation, cytokine release, or the activity of other immune effector cells [84].

With advances in research, the conceptualization of biomaterials is transitioning from “immune evasion” to “immune interaction” [85]. Understanding of the complex interactions between biomaterials and host immune cells within the tissue microenvironment is essential for designing therapeutic biomaterials that promote tissue integration while minimizing foreign body reactions (FBR). This section synthesizes how material properties influence immune cells—including the underexplored adaptive compartment—to inform the design of immunomodulatory biomaterials.

### Effect of biomaterial composition on the degree of immune response

**Figure 5:**
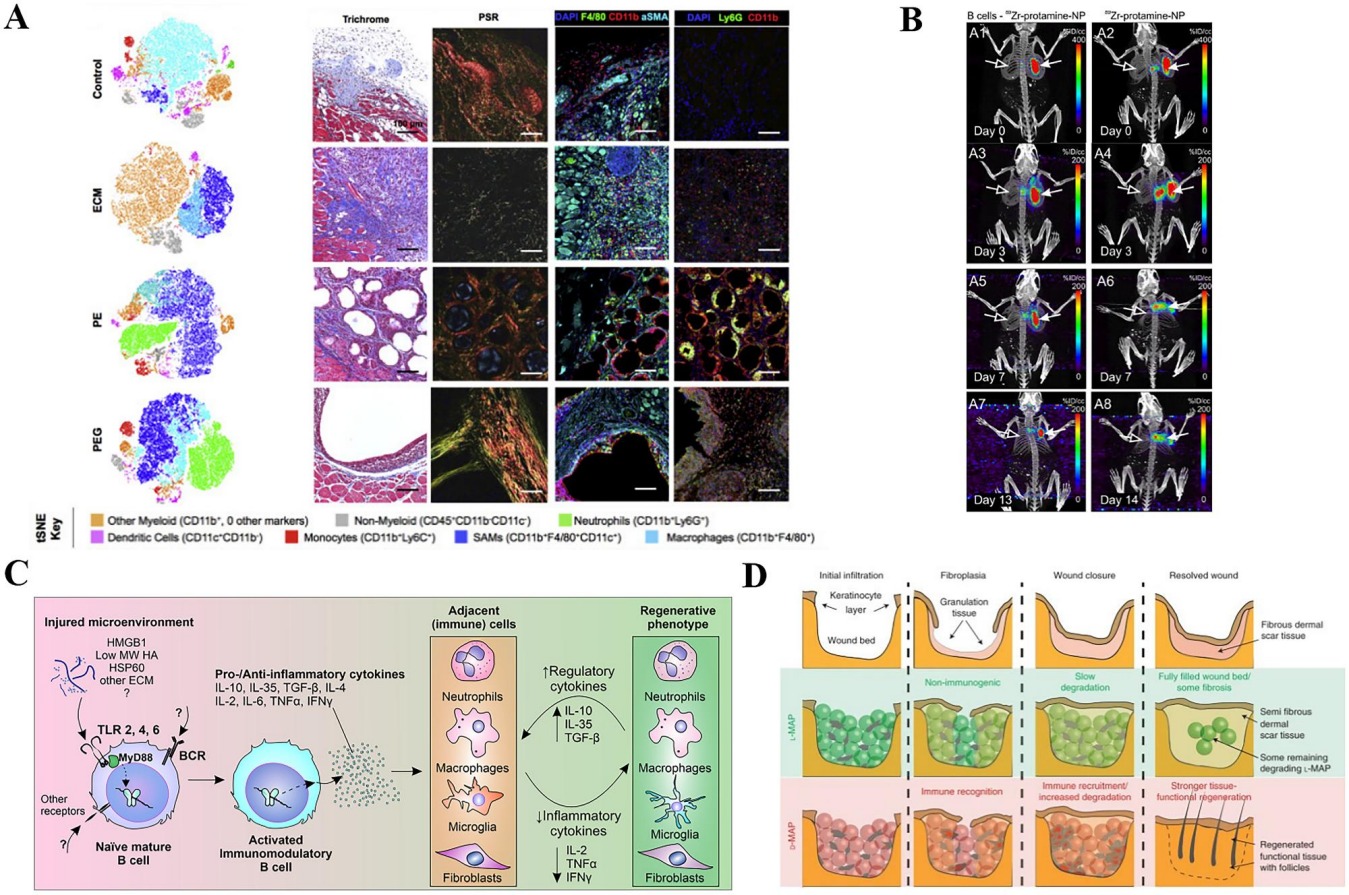
Biomaterials with different components trigger different levels of differential foreign body response (FBR). (**A**) Comparative immune profiling of natural versus synthetic scaffolds three weeks after injury, t-distributed stochastic neighbor embedding (t-SNE) analysis exhibits distinct immune cell clustering, with a higher degree of neutrophil infiltration in synthetic scaffolds. Histological assessment showed synthetic scaffolds exhibit immune cell distribution indicative of a stronger FBR. Reproduced with permission from Ref. [86]. (**B**) PET/CT tracking of B cells after their application to the wound bed demonstrates that mature B cells accelerate wound healing in both acute and chronic diabetic skin lesions. Reproduced with permission from Ref. [74]. (**C**) Indirect secretion of cytokine Cascades by B cells in the damaged microenvironment regulates tissue regeneration process. Reproduced with permission from Ref. [87]. (**D**) Immunological processes mediated by L-MAP and D-MAP promote tissue repair and regeneration. Reproduced with permission from Ref. [88].

The molecular composition of biomaterials provides the initiating signal for the immune repair cascade. Their chemical structure and degradation products determine the recognition patterns of APCs, thereby steering adaptive immunity toward either pro-inflammatory or tolerance phenotypes, and ultimately guiding repair toward fibrosis or regeneration. Biomaterials, including natural, synthetic, and composite materials, facilitate the recruitment of immune cells to tissue injury sites, with synthetic materials exhibiting a higher degree of neutrophil infiltration [86]. Three weeks post-implantation, synthetic scaffolds often exhibit immune cell distribution indicative of a stronger FBR [86] (Figure 5A). The mechanisms of immune activation also differ across material components [74, 87, 88] (Figures 5B–D). For instance, the collagen-glycosaminoglycan networks in decellularized matrices induce a microenvironment rich in IL-4/IL-10 and low in TNF-α, that promotes CD4^+^ T cell polarization toward Foxp3^+^ Tregs and M2 macrophage differentiation. This significantly accelerates vascularized regeneration in skeletal muscle defects [33]. Conversely, oligolactic acid released from degrading poly(lactic-co-glycolic acid) (PLGA) elevates local IL-17 levels via TLR4-MyD88 signaling, delaying diabetic wound healing. This suggests that material composition serves as a key regulator of immune modulation [89].

#### Natural materials

Natural materials such as collagen, hyaluronic acid (HA), and decellularized matrices are rich in arginine-glycine-aspartic acid (RGD)/adhesion sites [21]. Upon implantation, they can be recognized by dendritic cells (DCs) via receptors like integrin α2β1, inducing low expression of CD80/86 and secretion of IL-10 and TGF-β, thereby polarizing naïve CD4^+^ T cells into Tregs [33]. Expanded Tregs suppress excessive Th1/Th17 responses by secreting IL-35, TGF-β and through cell contact-dependent mechanisms, while simultaneously driving macrophage polarization from M1 to M2 phenotypes. This establishes an “anti-inflammatory-tolerogenic” microenvironment. This adaptive immune pattern significantly reduces IFN-γ and TNF-α levels while enhancing VEGF and platelet-derived growth factor (PDGF) expression, thereby promoting angiogenesis and fibroblast proliferation. The outcome is physiological tissue repair characterized by minimal scarring and vascularization [89, 90].

#### Synthetic materials

Synthetic materials, such as PLGA, polycaprolactone (PCL), or polyethylene glycol (PEG)-based hydrogels, can be engineered to program adaptive immunity through surface charge, topology, and controlled release. For example, positively charged microdomains that moderately upregulate DC-CD80/86, combined with release of IL-4 and CCL22, can precisely direct CD4^+^ T cell responses toward Th2/Treg lineages. Slow release of surface-adsorbed antigen-peptide major histocompatibility complex (MHC) complexes may further induce antigen-specific Treg expansion and suppress cross-reactive Th1/cytotoxic T lymphocyte (CTL) responses [91]. This “Th2-Treg synergistic” model limits chronic inflammation, upregulates IL-4/IL-13, promotes M2a macrophage activation, and continuously secretes Arg-1 and VEGF to support regeneration of bone, cartilage, or nerves. As the material degrades synchronously with tissue remodeling, it is replaced by an ordered ECM, achieving functional tissue reconstruction [92, 93].

In summary, material composition plays a critical role in dictating the magnitude and nature of the immune response. Selecting a material system therefore requires careful consideration of both its inherent properties and the specific context of the injured tissue.

### Material stiffness and bidirectional regulation of immune-cell polarization

**Figure 6:**
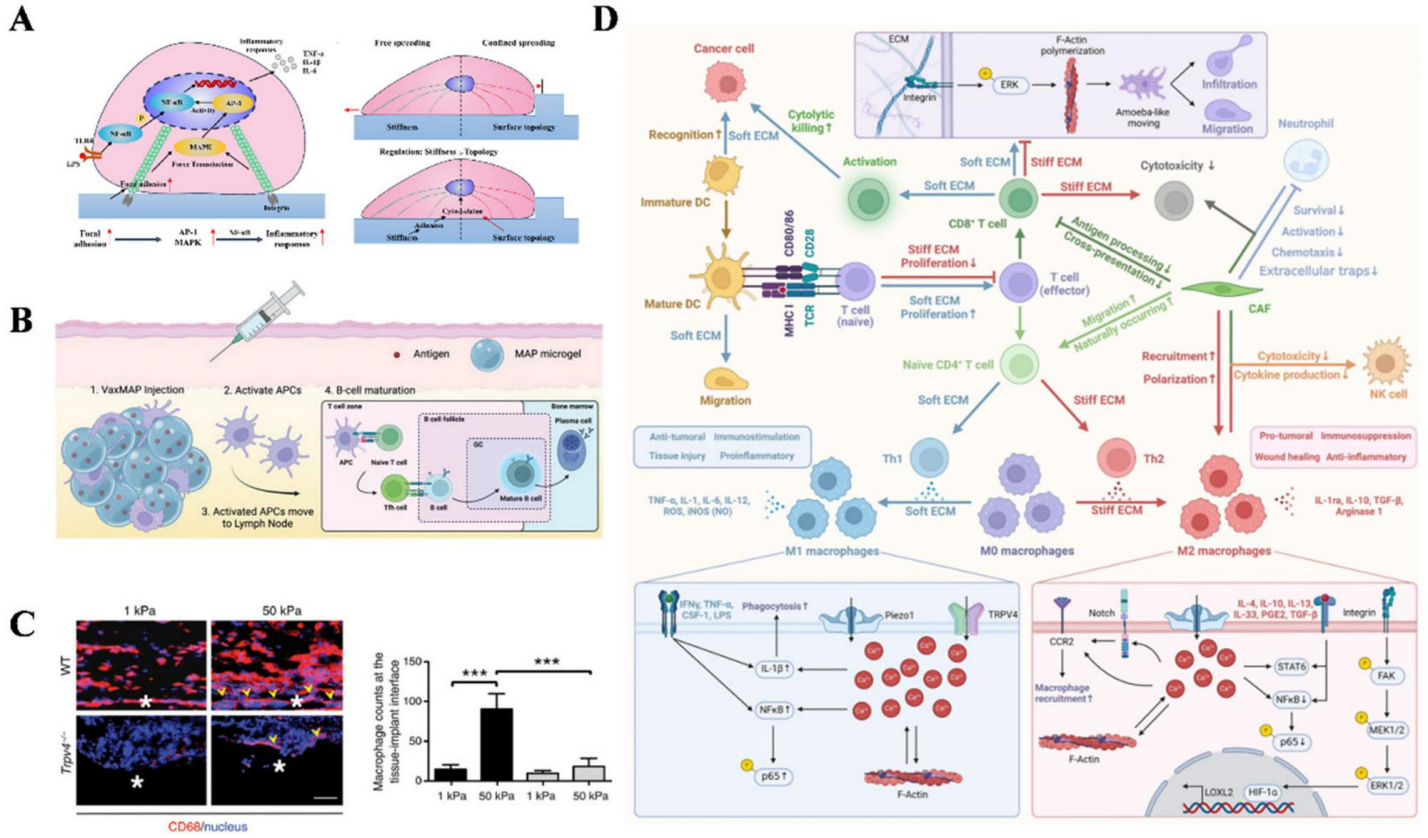
Biomaterial stiffness directs immune-cell interactions. (**A**) Potential intrinsic mechanisms of stiffness-mediated inflammatory responses in M1 macrophages. Reproduced with permission from Ref. [95]. (**B**) Material-guided adaptive immune responses. Gels degrade to release antigen which is uptake by antigen-presenting cells (APCs) to direct an adaptive immune response. This biomaterial-induced immune response is tunable by enhancing material stiffness through altering material cross-linking density. Reproduced with permission from Ref. [96]. (**C**) Material stiffness trigger macrophage accumulation on the surface of the implant. As an important hallmark of FBR, it increased nine-fold in WT mice receiving 50-kPa implants compared to 1-kPa implants. Reproduced with permission from Ref. [97]. (**D**) Overview of cellular mechanisms in immune cells influenced by ECM stiffness. A soft ECM environment enhances T cell proliferation, promotes CD8^+^ T cell activation and migration, and guides the polarization of naïve CD4^+^ T cells and microphages toward Th1 and M1 phenotypes. A stiff ECM has converse effect. Reproduced with permission from Ref. [107].

Cells sense mechanical force through material properties (e.g. material composition and stiffness), and convert these physical cues into biochemical signals via mechanotransduction pathways [94, 95] (Figure 6A). In ECM materials with different levels of stiffness, studies have detailed the cascade reactions and interactions of immune cells triggered by the materials [96, 97] (Figures 6B and C), highlighting the main mechanism by which the physical properties of a material can modulate biological functions.

Immune cells exhibit distinct stiffness-dependent responses. Macrophages are highly sensitive to mechanical forces, and their polarization can be directed by substrate stiffness [98, 99]. Mechanical stiffness can also control dendritic cell metabolism and function [100]. Similarly, T cell changes can also be modulated by substrate stiffness, with APCs providing antigens from physical or chemical signals to influence T cell fate [101]. This mechanosensing by T cells involves YAP to transfer mechanical signals to immune responses [102]. Material stiffness can also directly regulate adaptive immune cells. T cells exhibit enhanced expansion and differentiation in softer matrix environments [103]. It was reported that T cells cultured on softer modified polydimethylsiloxane (PDMS) substrates without exogenous cytokines showed a propensity for differentiating naïve CD4^+^ T cells into Th1 cells [104]. Contradictory findings indicate that stiffer matrices, resembling the mechanical properties of native bones, may enhance the differentiation and mineralization of MSCs [105, 106]. Overall, stiffness profoundly affects cellular behavior and immune response [107] (Figure 6D). Therefore, selecting a material with mechanical stiffness that aligns with the native tissue’s mechanical environment may be advantageous.

### Synchronization of material degradation characteristics with tissue regeneration rhythms

**Figure 7:**
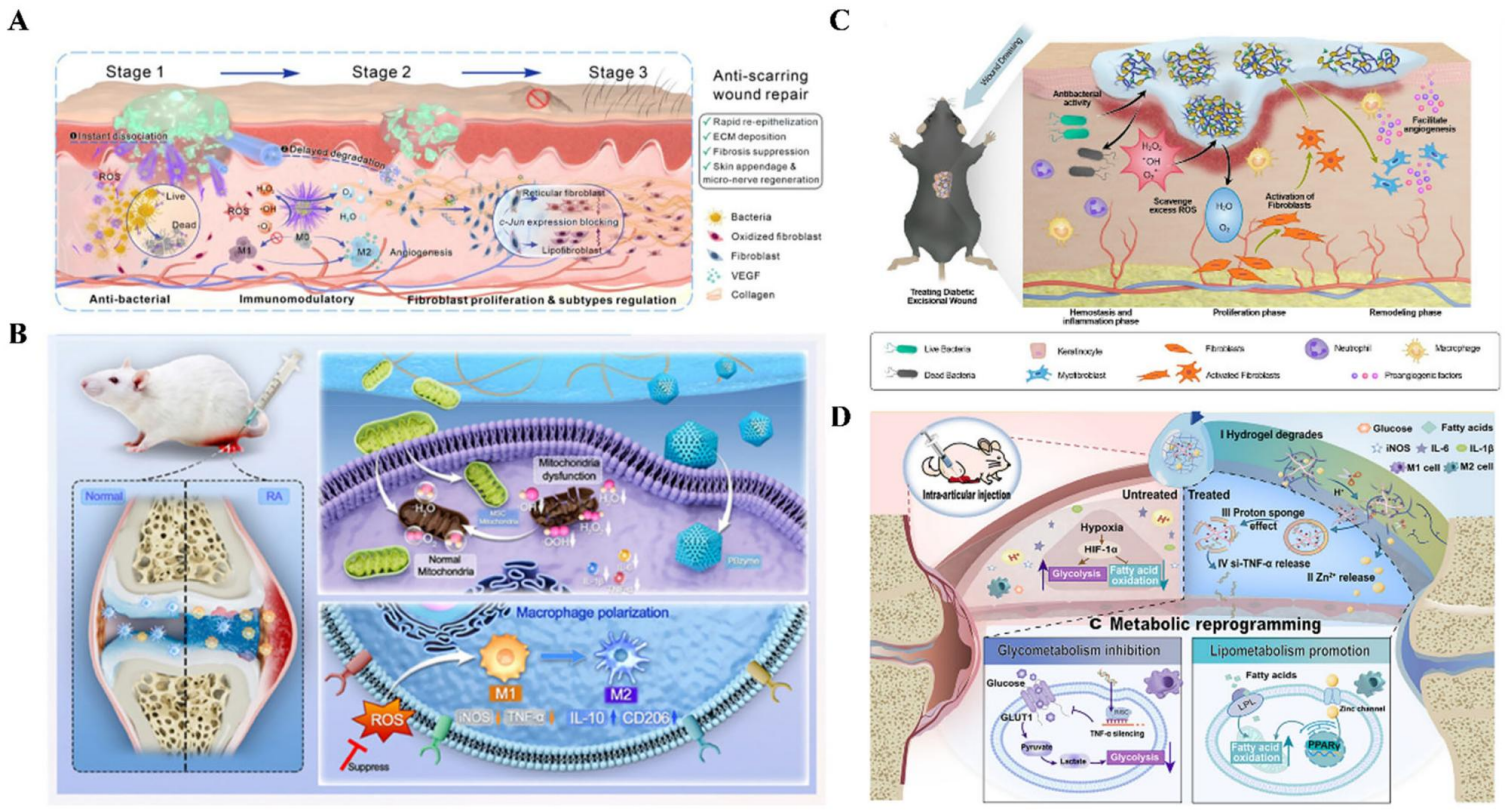
Adaptation of material degradation to regulate the microenvironment. (**A**) Mechanistic overview of F/R gels designed to facilitate scarless wound healing. This healing phase-adaptive regulating hydrogel (F/R gel) achieves programmed modulation of chronic infected wounds through hierarchically delivering performance triggered by hydrogel degradation. Reproduced with permission from Ref. [109]. (**B**) Mechanism of tunable-degradation hydrogels for rheumatoid arthritis (RA) treatment. A polymer-modified DNA hydrogel with finely tuned degradation rate enables the sustained support of bone and cartilage repair while controllably releasing therapeutic agents. Reproduced with permission from Ref. [110]. (**C**) Mechanistic insights into materials utilized for diabetic wound treatment. Ceria-nanoparticle-entangled reticulation realizes adaptive degradation synchronized with the healing phases to regulate local inflammation and immune responses. Reproduced with permission from Ref. [111]. (**D**) Mechanistic diagram of a degradable hydrogel modulating macrophages in metabolic reprogramming for the treatment of rheumatoid arthritis. Reproduced with permission from Ref. [112].

The degradation kinetics of biomaterials are now recognized as a key determinant of the immune-repair cascade. The degradation rate, chemical profile of degradation products, and release dynamics collectively determine the duration and intensity of the “danger” signal perceived by APCs [27]. In general, degradable natural polymer materials tend to release nontoxic compounds that are structurally similar to endogenous biomolecules, provide favorable cell adhesion sites, and cause a relatively lower degree of FBR [108]. Thus, the degradation properties of biomaterials should be carefully engineered to counteract chronic inflammation. For example, it was reported that a combined material is designed with time-sequential degradation release and immunomodulation for promoting tissue repair [109] (Figure 7A).

The degradation rate depends on intrinsic material properties (e.g. composition, degree of cross-linking, and mechanical properties) and the microenvironment [113]. Rates that are too fast or too slow compared to tissue regeneration can lead to undesirable consequences: incomplete healing, accumulation of degradation products, material encapsulation, and failure of tissue integration. The host response is influenced by both the degradation process and the ability of macrophages to clear foreign material [110–112] (Figure 7B–D).

Deliberate control of degradation rates through material design as well as an understanding of how these rates affect immune cells is therefore essential. ECM degradation products promote M2 macrophage polarization and tissue regeneration [114]. Lower-molecular-weight HA enhances DC activation and T cell proliferation [115]. Signaling pathways involved include NF-κB and MAPK in silk-gel hydrogel-induced polarization of macrophages toward an anti-inflammatory phenotype [116]. Studies tuning ester/amide bond ratios demonstrated that rapid degradation group (100% ester bonds) released large amounts of oligomers that stimulate DC maturation, induce Th1 dominance, cause persistent M1 macrophage infiltration, and disordered collagen deposition. In contrast, the slow-degradation group (25% ester bonds) showed increased Foxp3^+^ Treg numbers and elevated M2 macrophage proportions, enabling organized vascularization and low-scar dermal regeneration [117]. Overall, harmonizing degradation and tissue regeneration is thus critical, requiring nontoxic byproducts that are efficiently eliminated.

### Appropriate pore size supporting tissue repair

**Figure 8:**
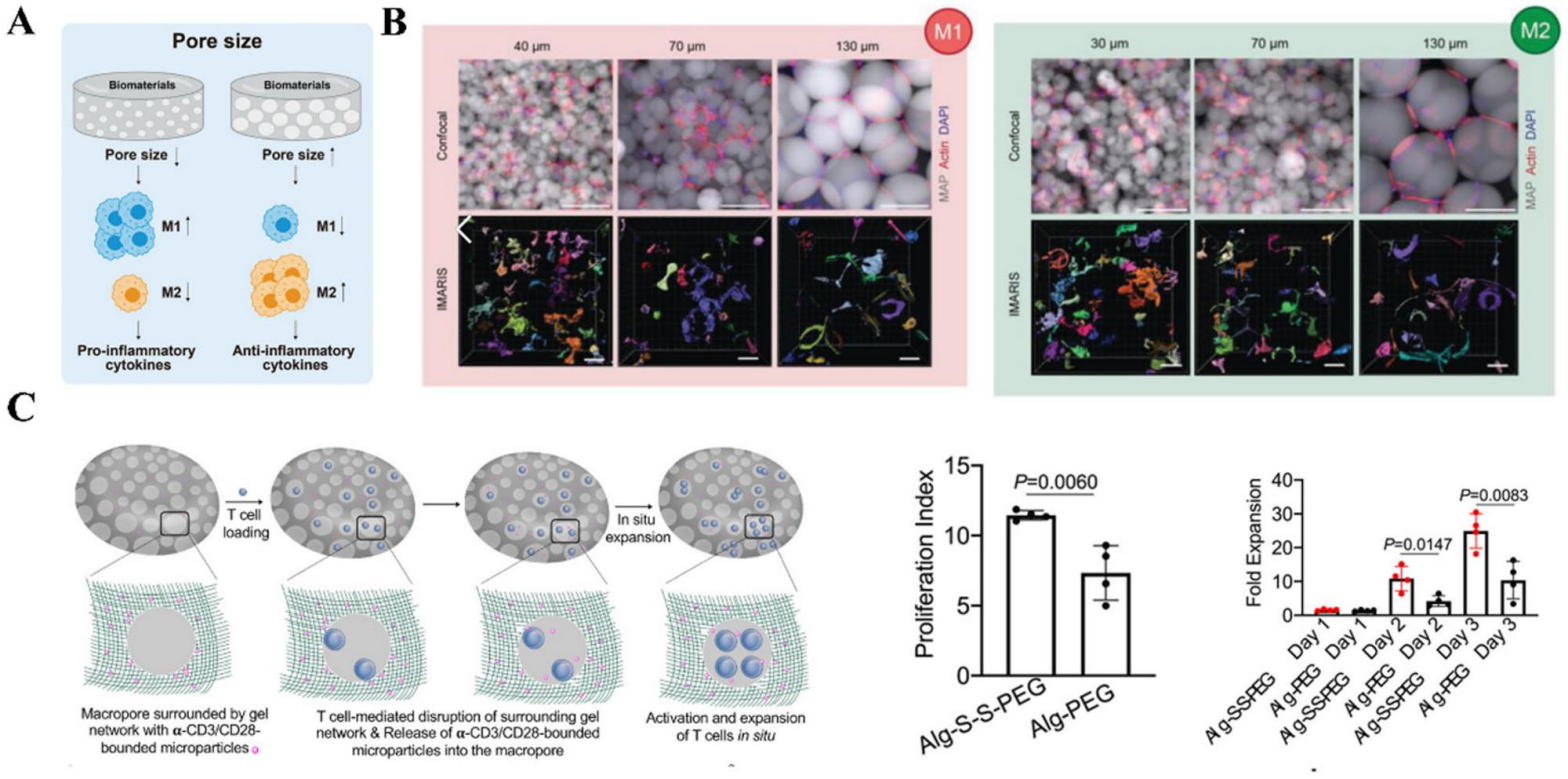
The role of appropriately sized pores in immunomodulation for tissue repair. (**A**) General principle of pore-dependent macrophage polarization. Materials with larger pore sizes promote the macrophage M2 phenotype. Reproduced with permission from Ref. [10]. (**B**) Spatial confinement tunes macrophage response. This is exemplified by research using microporous annealed particle (MAP) scaffolds with varied particles sizes (40, 70, and 130 µm) and found that in scaffolds with pore size on the scale of cells lead to a reduced level of the inflammatory response. Reproduced with permission from Ref. [119]. (**C**) Macroporous structure modulates adaptive immunity. An injectable T cell-responsive macroporous hydrogel enabled the expansion of functional T cells *in vivo* and improved cytotoxic T lymphocyte (CTL) response. Reproduced with permission from Ref. [126].

In tissue engineering, optimal pore size and high porosity are essential for cell adhesion, proliferation, differentiation, and nutrient/waste exchange [118]. Material pore size regulates the behavioral fate of immune cells. Materials with larger pore sizes promote the macrophage M2 phenotype [10, 119–122] (Figures 8A and B). For neutrophils, larger pore diameters reduce NETs formation on surfaces and promote implant integration to the tissue [123, 124]. Scaffolds with macroporous structure could entice effector T cells to modulate the immune environment [125]. A group fabricated an injectable T cell-responsive macroporous hydrogel that enabled the expansion of functional T cells *in vivo* and improved CTL response [126] (Figure 8C). Engineered nanoporous surfaces (20–400 nm) revealed that 200 nm pores significantly improve T cell signaling and activation [127]. However, conflicting results showed that larger-pore-size (160 μm) poly (hydroxyethyl methacrylate) hydrogels exhibited greater infiltration of M2 macrophages, whereas smaller-pore-size (34 μm) hydrogels led to increased vascular density [128].

Thus, an “optimal” pore size is closely related to the material composition and target tissue. Pore size gradient may be effective for the formation of multiple tissues and tissue interfaces. Appropriate pore sizes are beneficial in promoting tissue regeneration.

### Alleviating inflammation with hydrophilic materials

**Figure 9:**
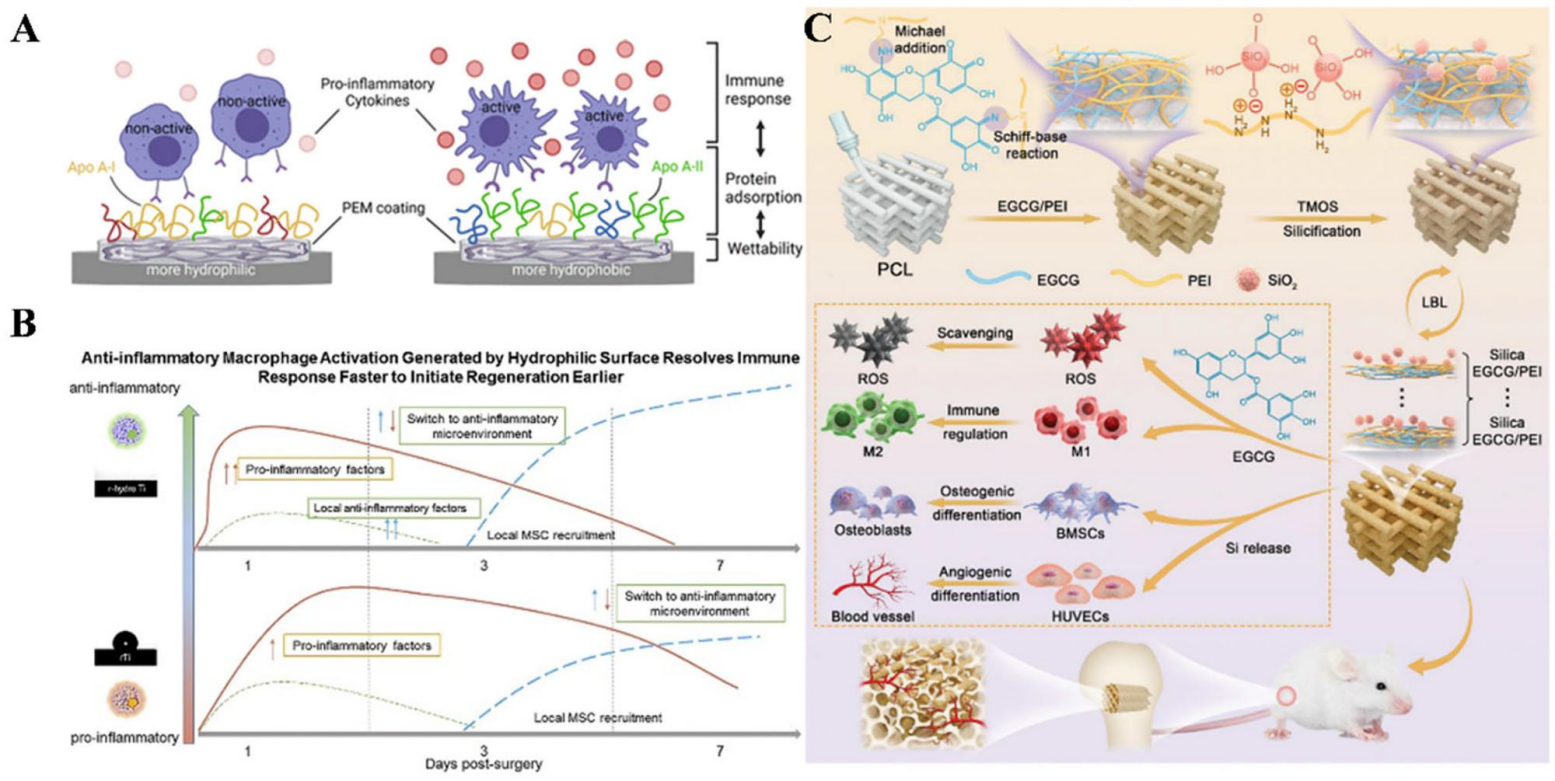
The influence of material wettability on immune-cell behavior and function. (**A**) The surface wettability of materials correlates with pro-inflammatory immune-cell responses and changes in protein adsorption. Hydrophobic surfaces adsorb inflammation-related proteins, triggering an increased expression of pro-inflammatory cytokines, while hydrophilic surfaces induce a mild immune response via anti-inflammatory protein adsorption. Reproduced with permission from Ref. [129]. (**B**) Surface wettability has an effect on both the innate as well as the adaptive systems. Increasing surface hydrophilicity on titanium implants induces macrophage polarization, triggers the adaptive immune response toward a pro-wound healing phenotype, and increases stem cell recruitment. Reproduced with permission from Ref. [134]. (**C**) The multifunctional coating strategy increases the surface hydrophilicity of the PCL scaffold and promotes immunomodulatory as well as osteo/angio-genic activity. Reproduced with permission from Ref. [135].

Surface wettability critically influences protein adhesion [129] (Figure 9A). Many biomaterials are hydrophobic that enhance protein binding and potential immunogenicity [130, 131]. Incorporating hydrophilic molecules is a common strategy to mitigate this phenomenon [132]. Hydrophilic and negatively charged surfaces could promote anti-inflammatory responses. Early research in biomaterial design focused on variations in wettability, revealing that hydrophilic surfaces facilitate tissue repair processes. Interaction with hydrophilic microrough titanium surfaces in bone tissue encourages macrophage polarization toward the M2 phenotype and enhances osteoblastic signaling [133]. Recent studies focused on the role of material wettability in the regulation of cell-cell interactions [134, 135] (Figures 9B and C). Wettability can influence the adsorption and conformational changes of biomolecules (such as immunoglobulins and complement proteins) on material surfaces, thereby affecting the activation of APCs like DCs and macrophages. For example, hydrophilic surfaces facilitate the exposure of cell-binding sites on fibronectin (FN), promoting integrin β1-mediated cell adhesion and M2 macrophage polarization [136]. Therefore, the activation status and antigen-presenting capacity of these APCs directly impact T cell activation and differentiation pathways. By optimizing material wettability, the local cytokine profile can be modulated to promote the release of anti-inflammatory cytokines. This guides adaptive immune cells toward tissue repair-favorable pathways and reduces chronic inflammatory responses [137]. Nevertheless, it has been proposed that hydrophilicity alone may not adequately predict the influence of materials on macrophage phenotypic polarization, suggesting that additional surface attributes might play a role in immune modulation [138].

### Complex regulatory networks of material surface properties on immune-cell functions

**Figure 10:**
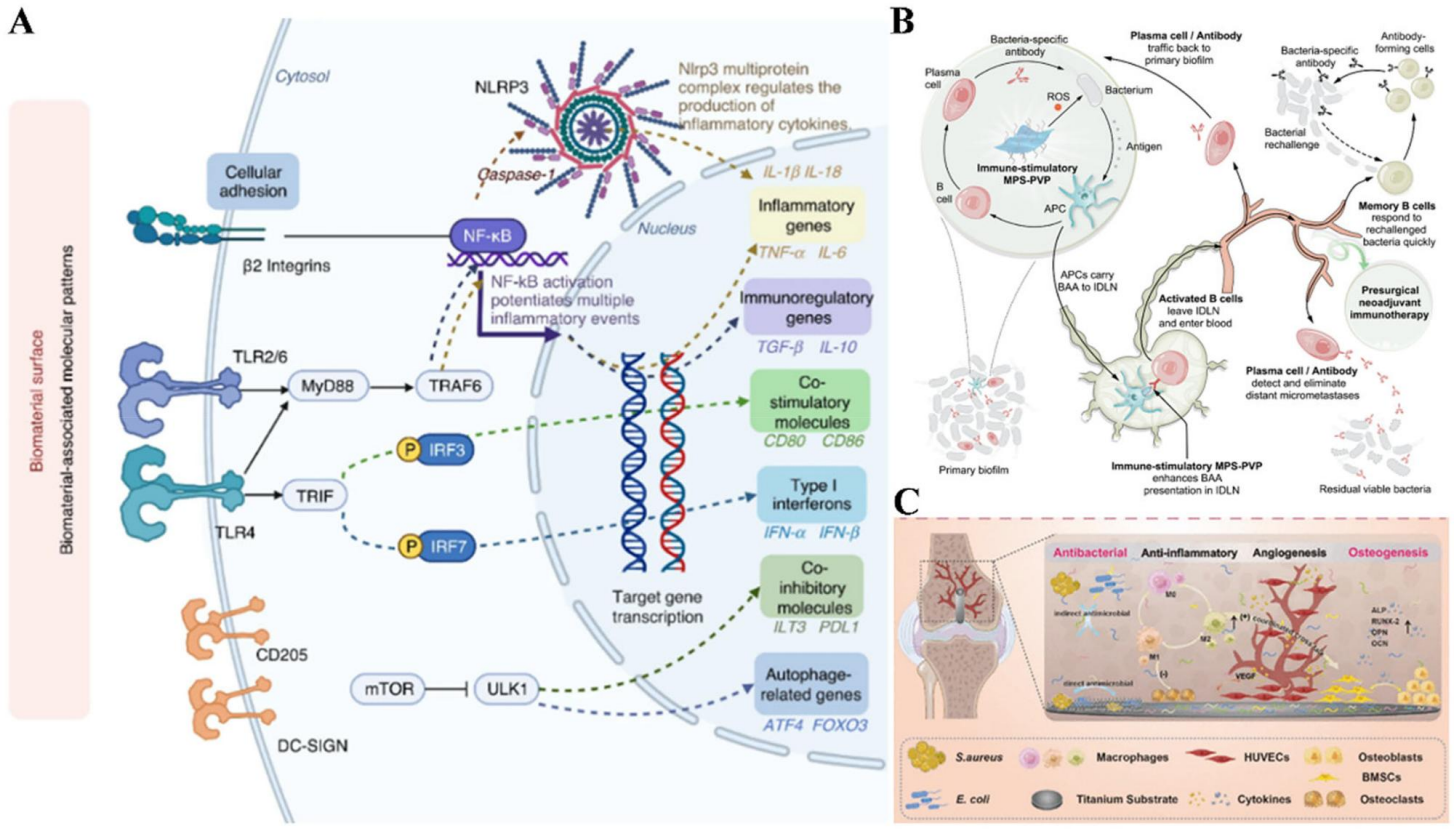
The surface chemistry of materials immunoregulates cellular complexity. (**A**) Mechanisms of dendritic-cell response to biological materials. This involves multiple mechanisms: β2 integrins adhesion initiating NF-κB signaling pathways, toll-like receptors (TLRs, TLR 2/4/6), C-type lectin receptors (CLRs, DC-SIGN and CD205), inflammasomes (nucleotide-binding oligomerization domain (NOD)-like receptor NLRP3) and autophagy. Reproduced with permission from Ref. [144]. (**B**) Immunostimulatory surface-modified biomaterials promote the activation and production of specific B cells and antibodies to enhance the immune response and prevent recurrence of infections. This process involves APCs carrying bacterial antigens trafficking to infection-draining lymph nodes to prime bacteria-specific B cells. Reproduced with permission from Ref. [145]. (**C**) Multifunctional surface modifications regulate complex material-cell interactions. Surface-modified titanium materials exert multiple regulatory effects to promote tissue regeneration, including antimicrobial, anti-inflammatory, pro-angiogenic, and osteoblastic effects, revealing immunomodulatory effect of tailored surface chemistry. Reproduced with permission from Ref. [146].

The surface chemistry of biomaterials can be altered through three primary methods: physical modification, chemical alteration, and radiation exposure. Detailed engineering strategies are reviewed elsewhere [139]. Common functionalization methods include grafting bioactive molecules (functional groups, cytokines) that alter the adsorbed protein layers, cell adhesion, surface charge, and wettability [140–142]. Surface coatings enhance long-term stability and biocompatibility of materials [143]. Therefore, a comprehensive understanding of how surface modifications impact immune cell behavior is vital for developing materials capable of modulating immune responses and promoting tissue repair [144] (Figure 10A).

Ongoing research continues to explore how the surface properties of materials affect the activation, proliferation, and responsiveness of adaptive immune cells. In one study, PDMS surfaces functionalization revealed that -COOH-modified surfaces significantly promoted CD4^+^ T cells activation and proliferation compared to -NH_2_ or -CHO modified groups. Additionally, both the -NH_2_ and -COOH groups preferentially directed the differentiation of naïve CD4^+^ T cells into Th1 cells relative to unmodified PDMS-Ab [104]. The activation of B cells and the subsequent antibody production triggered by the material surface can also play an immunomodulatory role in facilitating tissue repair [145] (Figure 10B).

The surface properties of materials are key regulators of cellular functions within complex material-cell interactions [146, 147] (Figure 10C). However, analyzing their effects is complex because these are associated with various properties. Changes in surface functional groups can alter wettability and surface charge, each of which may affect the immunomodulatory function of the material. Combining complementary material systems into composite materials is a viable strategy [148]. Thus, it is important to understand the relative contribution of each feature to immunomodulation. It is hoped that future research can identify the primary features or feature sets responsible for a material’s immunomodulatory effects. This knowledge would enable more targeted and effective material design, ultimately improving immunological guidance for tissue healing across different material types.

In summary, we provide a systematic overview of the pivotal role of biomaterials in modulating immune responses and the intricate interplay between material properties and the immune system. Given the extensive and complex influence of material physicochemical properties on adaptive immune outcomes, this review cannot comprehensively address all aspects; relevant research advances are summarized in Table 1.

**Table 1:** Summary of the effects of biomaterial properties on adaptive immune cell function.

Material properties Category	Immune cell	Physicochemical properties of biomaterials	Biological functions influenced by adaptive immune cells	Ref
Composition	CD8^+^ T cell	Alginate	Antioxidant gene expression↑, fibrosis↓	[149]
	B cell	B3-Cu-Zn-BG (Bioactive glasses)	Selective depletion of B cells, TNF-α secretion↓, osteogenic differentiation↑	[150]
	B cell	Alginate	B cell recruitment↑, fibrosis↑	[151]
	T lymphocytes	Nano-HA and nano-SiHA	T cell depletion↑, fibrosis↑, osteogenesis↓	[152]
	CD4^+^ T cell	MSCM-coated microribbon scaffolds	Treg/Teff ratio↑, local inflammation↓	[153]
	CD4^+^ T cell	ECM	Muscle fiber fusion↑, collagen deposition↓, muscle strength recovery↑	[154]
Stiffness	Mature DC cell	0.2–25 kPa PA gel	Stiffer substrate: IL-2 secretion↑, T cell proliferation↓	[155]
	CD4^+^ T cell	7.1 and 50.6 kPa PA gel	7.1 kPa PA gel: T cell activation↑	[156]
	CD8^+^ T cell	100–300 nm microparticle-based aAPCs	Cytotoxic T cell-dominant, without fibrosis or Th2 response	[157]
	Primary CD4^+^/CD8^+^ T cells	50 kPa–2.3 MPa PDMS	50 kPa PDMS: T cell expansion↑	[158]
	CD4^+^ T cell	0.2 kPa and 100 kPa polyacrylamide hydrogels	Soft polyacrylamide hydrogels: T cell proliferation↓, Th1/Th17 polarization↓, chronic inflammatory fibrosis↓	[159]
	CD4^+^ T cell	4 kPa and 40 kPa 3D scaffold matrices	T cell migration↑, IL-2 secretion↑, bone regeneration	[103]
Degradation	Treg cell	HMW-HA (>500 kDa)	Treg expansion↑, IL-10 and TGF-β secretion↑, chronic inflammation and fibrosis↓	[115]
	CD4^+^ T cell	Slow degradation 0% PEG-4eMAL and rapid degradation 100% PEG-4eMAL	Rapid degradation 100% PEG-4eMAL: IL-4 secretion↑, Th2 expansion↑, collagen deposition↑, osseointegration↑	[116]
Pore size	CD4^+^ T cell	40 μm PTS and 100 μm PTS	40 μm pores induce Treg polarization, suppressing Th1-mediated wound healing; 100 μm pores promote Th1-mediated inflammation	[160]
	DC cell	250–425 μm PLG	Promote DC maturation and antigen presentation to induce T cell-mediated antitumor immunity.	[161]
	DC cell	20/40/90 μm pHEMA/PDMS	20 μm pore size preserves the most activated DC-driven T cell immunity	[162]
	CD8^+^ T cell	50 nm CMPT	Th1 immune response	[163]
Hydrophilic	Treg cell	Hydrophilic rough Ti	Foxp3 and IL-10 expression↑, inflammation duration↓	[134]
	CD4^+^ Th2 cell	Hydrophilic surface	IL-4 secretion↑, Th2 expansion, collagen deposition↑	[136]

## Considering the use of biomaterials regulating crosstalk between innate and adaptive immune cells to better promote tissue repair

The immunomodulation mediated by biomaterials orchestrates the intricate interplay between innate and adaptive immune cells, thereby aiding tissue repair and regeneration. However, the current understanding of this process remains incomplete. This section presents recent insights into the interactions among immune cells influenced by biomaterials, aiming to clarify the effects of these materials on the immune system *in vivo*.

Under sterile conditions, non-degradable materials primarily activate the innate immunity, recruiting neutrophils and macrophages within hours post-implantation. Lacking micron or nano scale degradation products *in vivo*, they generally do not serve as a source of antigen for adaptive cells [164]. In contrast, degradable materials release fragments that are recognized by DCs and macrophages. These APCs process these antigenic signals and present them to T and B cells, thereby initiating adaptive immune responses [165, 166]. Monitoring of a fluorescently labeled degradable hydrogel revealed increased infiltration of APCs (macrophages and DCs) around the material, which triggered and directed adaptive immunity. They underscored the necessity of a long-term (∼21 days) assessment of the T cell immune response as a critical indicator of successful material integration [167].

Recent advancements have enhanced the understanding of how biomaterials mediate the interactions between innate and adaptive immune cells, particularly the communication between T cells and macrophages. It has been suggested that T cell responses to the wettability and roughness of biomaterials are modulated by macrophage activity, and the absence of T cell α-receptors may also influence the recruitment of MSCs. The synergistic action of αβ T cells and macrophages promotes MSCs recruitment, thereby facilitating bone regeneration. Furthermore, the Th1 response is frequently associated with M1 macrophage polarization, with Th1-derived cytokines, such as IFN-γ and TNF-α, driving this polarization. The interplay between Th1 and M1 cells amplifies a pro-inflammatory feedback loop [168]. The dynamic communication between T cells and macrophages is exemplified in experiments with Rag1^-/-^ mice, which lack mature T and B cells. In these studies, the upregulation of M2 macrophage markers was observed only after the Rag1^-/-^ mice were reconstituted with wild-type CD4^+^ T cells, leading to the restoration of M2 macrophages [91, 169]. Additionally, the polarization of T cells may depend on the innate immune cells present on the material’s surface, which can polarize into either pro-inflammatory or anti-inflammatory phenotypes upon recognizing the presented antigen. It has been demonstrated that an *in vivo* environment devoid of macrophages results in decreased T cell and MSCs recruitment, and T cell-mediated anti-inflammatory responses align with the presence of anti-inflammatory macrophages in homogeneous-material contexts [134, 170, 171]. In other words, T cells and macrophages modulate each other’s polarization states through a dynamic feedback mechanism involving soluble cytokines. The interplay between these two cell types is complex and influenced by various environmental factors, making it more intricate than a simple Th1/M1 paradigm. Research has aimed to design hydrogels that leverage coordinated T cell-macrophage interactions to activate pathways that promote tissue repair. Moreover, reciprocal crosstalk exists between B cells and macrophages. In studies that investigated how B cells regulate macrophages following biomaterial implantation using B cell knockout mice, reduced fibrosis around subcutaneous implants was shown, along with a significant decrease in macrophage presence at the material interface [172]. This indicates that B cells and macrophages communicate with each other, influencing the fibrotic response to the material. B cells also impact macrophages through soluble cytokines and secreted antibodies [151].

In conclusion, it is vital to emphasize the importance of adaptive immunity and to evaluate the long-term compatibility of biomaterials. Sole reliance on innate immune responses is inadequate for outcome prediction, and a comprehensive understanding of adaptive immunity in relation to biomaterials is expected to enable timely interventions for adverse events. Therefore, elucidating the intricate interactions between innate and adaptive immunity is essential for enhancing materials designed for tissue repair and regeneration.

## Modulation of immune response by artificial-intelligence screening materials

**Figure 11:**
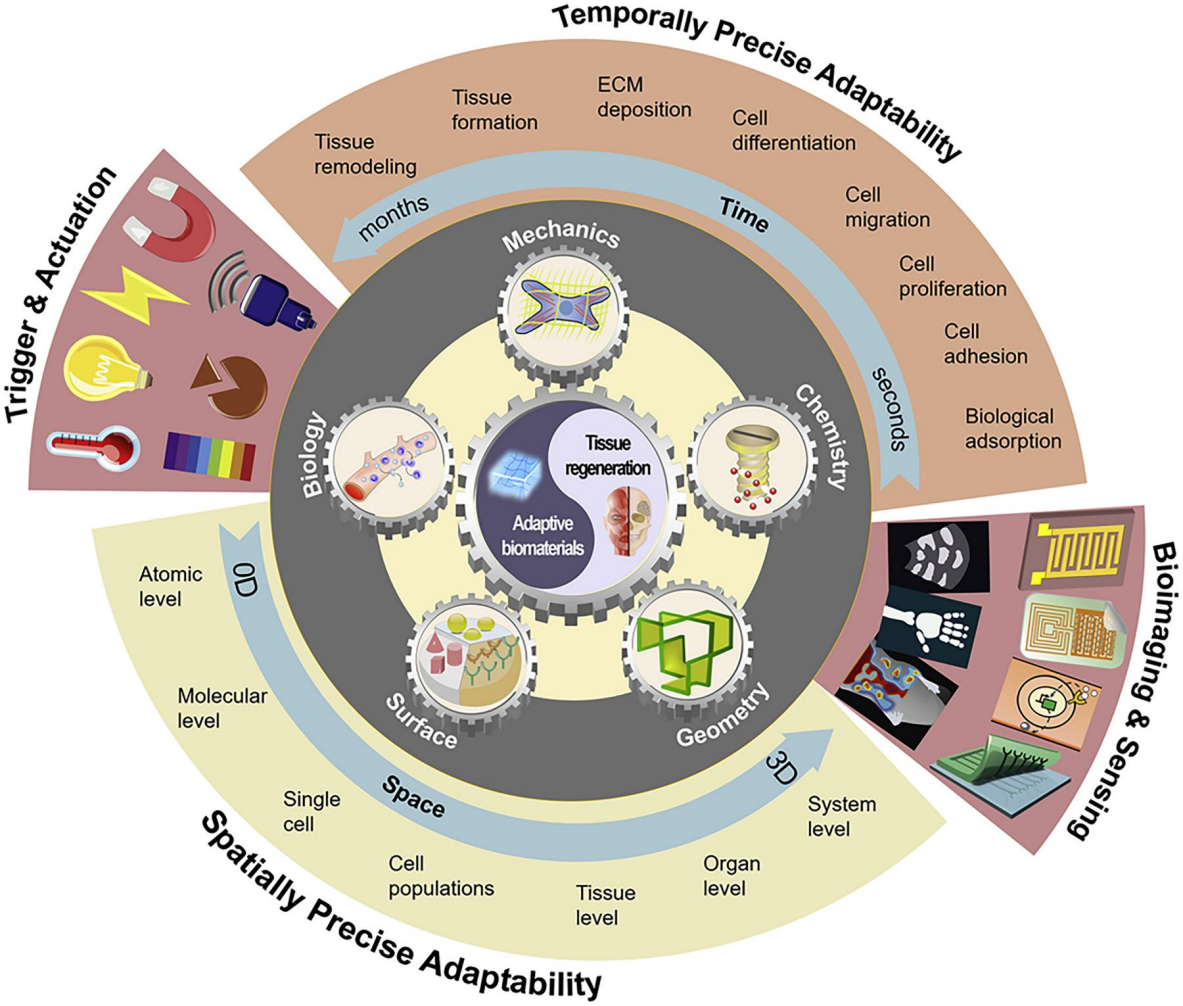
The principle of precise bioadaptability describes biomaterials capable of dynamic, spatiotemporally specific interactions with host to enable precision regenerative medicine. Such biomaterials are engineered in biology, mechanics, chemistry, surface, and geometry to dynamically and actively respond to either endogenous biological milieus/signals or external triggers with both spatial and temporal precision, ranging from atomic, molecular, and cellular regimes to tissue, organ, and system levels and within time frames spanning from seconds to months and years. Exogenous stimuli could augment the material bioadaptability, while *in situ* bioimaging or biosensing enables feedback-responsive theranostics and regeneration. Reproduced with permission from Ref. [174].

It is well known that the development of traditional biomaterials is lengthy and costly. With the development of artificial intelligence (AI) and the increase in data volume, researchers have attempted to apply AI models to the field of materials science. AI has demonstrated an excellent ability to process and analyze big data in recent years, and its expanding scale and applications have attracted widespread attention for better integration and processing of cross-disciplinary data relationships. AI plays an important role in constructing an integrated materials “gene pool,” optimizing biomaterial design and predicting interactions with tissues. The widespread use of AI is anticipated to significantly reduce development cost through trial-and-error minimization. It also holds promise for yielding effective therapeutic approaches and innovative treatment strategies [173]. Guiding principles for achieving precise biological adaptation through such methods have been proposed [174] (Figure 11).

To better utilize AI’s ability to process data, it is necessary to first create a comprehensive database of materials. Li *et al*. [175] created a database of hydrogels based on different assemblies of peptide structures containing more than 2000 peptides and used machine learning (ML) to predict the association between peptide structures and hydrogel properties. ML can also assist in the design and optimization of biomaterials that have been applied in wound healing and tissue engineering. Papa *et al*. [176] used a combination of *in vitro* experiments and AI modeling to determine the mechanical properties of the materials.

AI can also optimize the distribution of tissue protease-cleavable (TPC) particles at the osteochondral interface, thus enabling personalization for different patients and accelerating the solution to a major clinical problem [177]. Shifting to a data-driven paradigm, AI uses descriptive-predictive-normative methods and large-scale data analysis to optimize the search for the most effective biological materials, demonstrating a strong driving force [178]. However, several challenges currently limit the application of AI in this field. Key issues include a lack of standardized protocols for experimental data collection [179] and the absence of established criteria for validating predictive models [173]. Furthermore, the lengthy clinical trial and regulatory approval processes present additional hurdles [180]. Inaccuracies at any stage of data generation or processing can lead compromise AI training and lead to erroneous predictions.

## Conclusion and outlook

In summary, the underlying cause of delayed tissue healing is uncontrollable inflammation. This chronic inflammatory state disrupts immune cell metabolism and function, ultimately destabilizing the microenvironment at the injury site. The outcome of tissue repair is heavily influenced by the dynamic crosstalk between innate and adaptive immune cells, particularly the T and B cells. In this cascade, adaptive immune responses are frequently initiated and shaped by signals from the innate immune system, a dependency that biomaterial design must account for. Although these adaptive immune cells constitute a minor fraction of the total cellular population, their regulatory role in regenerative medicine is crucial.

Current material-based strategies have shown considerable promise for modulating immune responses to promote tissue repair and regeneration. Biomaterials can function as immunomodulators by controlling their surface physicochemical properties. Various aspects of materials, including composition, stiffness, and degradation, play unique roles in modulating immune cell behavior. A synergistic combination of these properties is essential for effectively guiding the repair process. Therefore, rational integration of material properties and deeper understanding of biomaterial-immune cell interactions are vital, especially the long-term compatibility of materials guided by adaptive immune cells. Despite this progress, research specifically targeting the effects of biomaterials on adaptive immunity remains limited. A more thorough evaluation of long-term compatibility of immunomodulatory biomaterials is required to fully harness the regenerative functions of adaptive immune cells. Advancing our understanding of complex interplay between highly versatile biomaterials and the intricate immune microenvironments will depend on leveraging advanced technology. Tools such as single-cell sequencing and AI modeling can provide referable suggestions to guide the design of next-generation immunomodulatory biomaterials. The absence of reliable data on material-driven T and B cell responses—specifically regarding dose-response kinetics, clonal tracing, and validation in chronic wound—constrains current conclusions. Present findings highlight important trends but fall short of establishing definitive mechanistic or clinical conclusions. Future work should therefore prioritize generating high-quality datasets to bridge these gaps and accelerate the development of clinically effective immunomodulatory therapies.
